# Patient journey mapping in an open-door psychiatric inpatient unit

**DOI:** 10.3389/fpsyt.2026.1882246

**Published:** 2026-07-20

**Authors:** Jorge Cuevas-Esteban, Esther Ruiz-Garros, Encarnacion Romero-Diaz, Nicole Motta-Rojas, Beltran Jimenez-Fernandez, Aurea Fernandez-Ribas, Barbara Martinez-Cirera, Anna Moreno-Orea, Laura Miro-Mezquita

**Affiliations:** 1 Departament Psiquiatria, Universitat Autònoma de Barcelona, Barcelona, Spain; 2 Servei Psiquiatria, Hospital Universitari Germans Trias i Pujol, Badalona, Spain; 3 Centro de Investigacion Biomédica en Red de Salud Mental (CIBERSAM), Madrid, Spain; 4 Institut Germans Trias i Pujol, Badalona, Spain; 5 NURECARE-IGTP Nursing Research Group, Institut Germans Trias i Pujol, Badalona, Spain; 6 INEDIT Research Group on Innovation, Health Economics and Digital Transformation, Institut Germans Trias i Pujol, Badalona, Spain

**Keywords:** mental health care, mixed-methods study, open-door policy, patient experience, patient journey mapping, psychiatric hospitalization

## Abstract

**Background:**

Open-door policies in inpatient psychiatric care have been increasingly adopted to enhance patient autonomy and reduce coercive practices. However, systematic methods for evaluating patient experiences in these settings remain limited. This study applied Patient Journey Mapping in an acute psychiatric unit operating a structured, time-limited (6 hours/day) open-door policy to comprehensively analyze the hospitalization experience.

**Methods:**

An exploratory sequential mixed-methods design was used. Qualitative data were first collected through focus groups with patients (n = 18), following GRAMMS guidelines, to identify critical touchpoints across the hospitalization journey. Quantitative data were then collected from patients (n = 32) using an adapted Likert-type ordinal scale to assess satisfaction with the identified touchpoints. Data were analyzed using content analysis methods.

**Results:**

The analysis delineated the main phases of hospitalization and identified key experiential touchpoints, including “Moments of Truth,” “Moments of Pain,” and “Wow Moments,” which substantially shaped patients’ perceptions of care quality. Open-door policies were perceived as supporting autonomy and reducing psychological distress. However, several challenges remained, particularly regarding the admission process, privacy during medication administration, and communication about structured activities.

**Conclusion:**

Patient Journey Mapping provides a useful patient-centered approach for evaluating experiences in open-door psychiatric inpatient settings. The findings highlight opportunities to optimize service delivery, strengthen therapeutic environments, and support recovery-oriented practices in acute psychiatric care.

## 1 Introduction

In recent years, mental healthcare has undergone a paradigm shift toward patient-centered models, prioritizing the reduction of coercive interventions and the promotion of recovery-focused care (1). Traditional closed-door policies in psychiatric inpatient units, historically justified as safety measures (2), have been linked to critical issues such as increased patient distress (e.g., perceptions of powerlessness and stigmatization) (3, 4), restricted autonomy (e.g., limited participation in care decisions) (3, 5), and lower satisfaction with treatment (6). For instance, locked environments in dual-diagnosis units correlate with higher patient agitation and reduced therapeutic engagement (5), while surveys reveal that both patients and staff associate closed-door practices with heightened tension and mistrust (6, 7).

Conversely, open-door policies in psychiatric settings have shown to promote therapeutic alliances, decrease incidents of seclusion and forced medication (8, 9), and enhance patient-reported outcomes, such as perceived dignity and collaboration in care (10). Empirical evidence from randomized trials, such as the Norwegian study by Indregard et al. (10), demonstrates that open-door approaches maintain safety standards while reducing coercive measures. Similarly, longitudinal implementations of these policies report sustained reductions in seclusion rates over six years (9). However, the implementation of open-door psychiatric units presents unique challenges, particularly in balancing safety with the promotion of patient autonomy and dignity (11). Patients generally view open-door policies positively, as they offer a sense of autonomy and reduce feelings of stigmatization (12). The open-door concept is often seen as a therapeutic tool that promotes patient-centered care. Patients appreciate the opportunity to engage in meaningful activities and interact with peers, which can foster a sense of community and support (11, 13).

Patient Journey Mapping has emerged as a methodology that helps healthcare professionals deeply understand the patient experience by visually mapping their path through the healthcare system. By identifying critical touchpoints, both positive and negative, throughout the care process, this mapping technique enables healthcare providers to design interventions that enhance patient engagement, improve service delivery, and support organizational change (14, 15). This approach integrates qualitative and quantitative data to highlight pain points, uncover opportunities for improvement, and facilitate the co-design of patient-centered interventions (14, 16). As highlighted in recent work by Sijm-Eeken et al., there is currently no universally accepted methodology for conducting patient journey mapping, and substantial heterogeneity exists in the rigor and quality of the approaches employed across studies (17). While the mapping of patient journeys facilitates the identification of barriers, enablers, and service delivery gaps from a patient-centered perspective, major shortcomings in the reporting of these initiatives continue to limit both transparency and replicability (18).

Despite growing recognition of patient experience as a key quality indicator in mental health services, structured approaches for its systematic assessment in acute inpatient settings remain scarce (3, 19). Self-reported satisfaction surveys, while widely used, often lack the granularity to capture the emotional and relational dimensions of hospitalization (20). In this context, Patient Journey Mapping offers a complementary approach by combining experiential narratives with structured patient ratings to identify critical touchpoints across the care process. Although this method has been applied in adjacent psychiatric contexts, such as emergency department boarding (21), we identified no prior studies applying it to the full hospitalization trajectory within an open-door acute psychiatric inpatient unit. This study therefore aimed to explore and map the patient experience in this setting using a mixed-methods approach. Specifically, the objectives were to identify the main stages and touchpoints of hospitalization, describe patients’ perceptions of these experiences, and generate a patient journey map to inform service improvement.

## 2 Methods

### 2.1 Setting

This study was conducted in 2022 at the open-door inpatient psychiatric unit of Germans Trias i Pujol University Hospital in Badalona, Barcelona, Spain. This facility is a general multispecialty hospital affiliated with the Autonomous University of Barcelona. At the time of the study, the GTUH served a catchment area encompassing 350,530 adults, distributed among four community mental health zones within the North Barcelona health district. At the time of the study, the acute inpatient psychiatric unit of the hospital functioned as a single ward with an operational capacity of 14 beds, staffed by a multidisciplinary team that includes senior and junior psychiatrists, nurses, psychologists, and social workers. The unit delivers comprehensive acute care for individuals with severe mental illnesses, utilizing a combination of pharmacotherapy, psychotherapy, social work, occupational therapy, and recovery-focused programs. Since 2019, the unit has progressively implemented the Safewards care model. Safewards is a set of ten interventions designed to enhance safety by preventing conflict and containment (22). Electroconvulsive therapy was available at the time of the study. Most patients (90-95%) are admitted from the emergency room. Patients typically present with severe primary psychiatric disorders characterized by acute disturbances and/or a risk of self-harm. The remaining individuals are referred by community mental health services. Of the participants included in the study, 60% were admitted involuntarily under applicable mental health legislation, while 40% were voluntary admissions. At the time of the study, the unit operated with an approximate staff-to-patient ratio of 1:7 during daytime shifts and 1:10 during night shifts.

The unit implemented a structured open-door policy in which the ward doors were opened for 6 hours per day, distributed between morning and afternoon periods, allowing patients to move freely outside the psychiatric unit within the hospital floor. Outside these designated periods, movement was restricted to the psychiatric ward. This approach had been in place since October 2021 and was consistently maintained throughout the week during the study period.

### 2.2 Design

This study employed an exploratory sequential mixed-methods design (23) to comprehensively map the patient journey in an open-door psychiatric hospitalization unit implementing the Safewards care model. In this design, the qualitative phase was conducted first and served an informing function: the themes, stages, and critical touchpoints identified through focus group analysis directly shaped the structure and item content of the quantitative satisfaction scale used in the subsequent phase. Both data streams were then integrated at the interpretation stage to produce the final Patient Journey Map (PJM), in which qualitative narratives and quantitative scores are presented in parallel for each hospitalization stage. This approach provided a comprehensive understanding of the patient trajectory from initial contact with the mental health unit through to discharge, capturing both overarching patterns and experientially rich detail.

Before conducting this mixed study, a multidisciplinary team of mental health hospitalization experts, including one psychiatrist, two psychiatry residents, one social worker, one psychologist, one nurse, and one auxiliary nurse, defined the starting, middle stage, and ending stages and moments that constitute the patient’s experience during mental health hospitalization in the open-door unit. They identified fifteen distinct moments grouped into five stages.

#### 2.2.1 Qualitative phase

To analyze patients’ perspectives, two focus groups with patients (total n=18) from the unit were conducted, each one divided into two sessions lasting approximately one hour (total of 4 hours). The number of participants was determined by the total number of patients available and willing to participate at the time of the study. The participants in the same focus group sessions remained largely consistent, with minor variations due to scheduling conflicts, and were held at the hospital facilities. The focus groups were integrated into the unit’s regular group routines; however, participants were explicitly informed that participation in the research was separate and distinct from their usual therapeutic activities. Information about participants and the duration of each session can be found in **Table 1**. The inclusion criteria for participants were as follows: be a patient admitted to the hospital’s acute mental health unit, be over 18 years old, and basic proficiency in Spanish or Catalan to understand the interview questions.

**Table 1:** Composition of the focus groups: session duration, number of participants, sex and age.

Focus group	Session	Duration (min)	Participants, n	Female, %	Mean age, years
Group 1	Session 1	60	10	60	40.4
Group 1	Session 2	45	8	40	44.0
Group 2	Session 1	60	8	25	56.5
Group 2	Session 2	60	8	40	52.0

Each focus group met across two sessions; the participants within a group were largely the same in both of its sessions, with only minor variation due to scheduling. Rows therefore report attendance per session, not unique individuals. The total number of unique participants was 18 (Group 1, n = 10; Group 2, n = 8).

Female, percentage of attendees in each session who were female. Mean age is given in years; session-level standard deviations were not recorded and are therefore not reported.

A total of 28 patients hospitalized in the unit during the study period were approached in person and invited to participate in the study; 10 declined to participate. Clinical staff determined that the patients’ current psychopathological status, including active psychotic symptoms, severe cognitive disorganization, or marked agitation, precluded the capacity to provide informed consent or engage meaningfully with the group dynamic. This selection process is acknowledged as a potential source of sampling bias.

Given that the stages and moments of the patient journey had been predefined by the expert panel, the aim of the qualitative phase was not to achieve theoretical saturation in the grounded theory sense, but rather to confirm, refine, and enrich the content of those predefined categories through patient perspectives. The number of participants was considered sufficient for this confirmatory exploratory purpose within a purposefully bounded sample (24).

Verbal informed consent was obtained from all participants after they were provided with detailed information about the study’s objectives, procedures, confidentiality measures, and their right to withdraw at any time without affecting their care. Participation remained entirely voluntary. The study adhered to ethical guidelines, receiving approval from the local research ethics committee (PI-25-133).

The interviews were conducted by a psychiatrist (JCE), who was not involved in the patients’ clinical care, and a social worker from the multidisciplinary team, who served as the note-taker. No other individuals were present in the interview room except for the participants and the interviewers. Interview guides were developed based on a literature review and refined after initial interviews to ensure relevance and capture detailed experiences. To effectively capture and visualize the patient’s journey, the dynamics of the sessions were centered around answering open-ended questions, “What does the hospital do well?” and “What does the hospital do poorly?” for each moment identified by the expert panel. Visual tools were used to help clarify the questions and guide participants’ responses at each stage. Moments and stages previously defined by the expert panel were refined during the focus groups according to patients’ opinions. Direct quotes from the participants were collected to enrich the data.

#### 2.2.2 Quantitative phase

After completing the qualitative phase of the study in March 2022, the research progressed to the quantitative phase. To evaluate patients’ subjective experience across key touchpoints of the care process, a structured instrument was developed by the research team based on an adapted Likert-type ordinal scale (25). The instrument was developed through a three-step process (1): item generation, in which the research team derived satisfaction items for each of the fifteen hospitalization moments based on the PJM framework and findings from the qualitative phase (2); content validity review, in which all items were reviewed by the full multidisciplinary team for clinical relevance and comprehensibility; and (3) a brief cognitive debriefing with three patients not included in either study phase, who confirmed item clarity. The final instrument was organized into two subscales: ‘Experience in Interactions’ and ‘Empathy and Professionalism of Staff.

The questionnaire used emotionally labeled response options ranging from highly negative to highly positive descriptors, from 1 (“I hate it”) to 6 (“WOW”), to capture not only satisfaction levels but also the emotional intensity associated with patients’ perceptions at each stage of hospitalization. This approach was informed by the semantic differential technique, which uses evaluative language to elicit connotative meaning (26), and aligns with patient-centered research, service design, and user experience approaches, where emotionally enriched response formats may help capture complex subjective experiences such as vulnerability, empathy, or perceived professionalism in healthcare interactions. Given the exploratory and complementary role of this phase within the mixed-methods design, and the pragmatic sample size constraints of a single inpatient unit, formal psychometric validation was not undertaken.

Participants completed the questionnaire prior to hospital discharge at the hospital facilities. The general inclusion criteria were identical to those of the qualitative phase, except that patients who had participated in the focus groups were excluded. Eligible participants received detailed face-to-face information about the study’s purpose and scope during a preliminary session, and written informed consent was obtained from all participants. Verbal informed consent was used in the qualitative phase because the research ethics committee waived the requirement for written consent for acutely hospitalized participants (PI-25-133); in the subsequent quantitative phase, which was conducted closer to discharge when participants were more clinically stable, written informed consent was feasible and was therefore obtained. A total of 32 assessments were conducted by a psychiatrist and two psychiatry residents, with an average duration of 20 minutes per assessment. The mean participant age was 45.2 years (SD = 16.7; range 19–76), and 18 of 32 participants (56.3%) were female. The sample size was determined pragmatically based on the number of eligible and consenting patients during the study period.

### 2.3 Data analysis

The qualitative data were analyzed using directed content analysis (27), an approach suited to studies where an *a priori* framework exists, in this case, the five hospitalization stages and fifteen moments predefined by the expert panel. Focus group recordings were transcribed verbatim and independently coded by two members of the research team (JCE and NMR), using the hospitalization stages as primary organizing units. Within each stage, open codes were generated inductively from the data, capturing specific experiential elements that either enhanced or detracted from the patient experience. These initial codes were reviewed in iterative consensus meetings, during which discrepancies were resolved through discussion and similar codes were merged into thematic categories. Inter-rater agreement was not formally quantified, but all disagreements were resolved by consensus before proceeding.

The three critical moment classifications, “Moments of Truth”, “Moments of Pain”, and “Wow Moments”, emerged from the integration of qualitative codes with quantitative scores. Drawing on experience-based design, Moments of Truth were defined as pivotal touchpoints shaping the patient experience (28), while Moments of Pain referred to problematic stages of the pathway, consistent with patient journey mapping approaches used to identify burdensome care transitions (29). Stages with convergent negative qualitative content and low satisfaction scores were classified as Moments of Pain, stages with high qualitative endorsement and high scores as Wow Moments, and stages identified as pivotal to the overall experience regardless of valence as Moments of Truth. **Table 2** presents the definitions of these moment classifications and the corresponding symbols used in the PJM.

**Table 2:** Definition of key moments of the patient experience during hospitalization and their symbolic representation in the PJM.

Key moments	Definition	Symbol
Moments of Truth	Critical points of contact between the hospital and the patient that significantly shape the overall hospitalization experience.	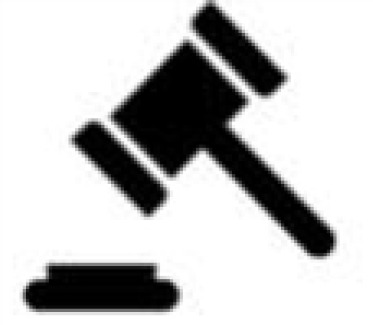
Moments of Pain	Situations that arise from negative interactions, leading to feelings of frustration, anxiety, or helplessness.	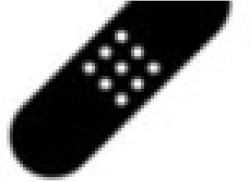
Wow Moments	Experiences in which a patient’s expectations are not only met but exceeded, creating a sense of delight and excitement.	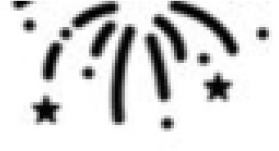

For the quantitative data, descriptive statistical analyses were then performed, including the calculation of means and standard deviations for each stage of the hospitalization process, providing objective measures of patient satisfaction. This quantitative analysis offered a detailed understanding of patient experiences, identifying specific stages where patient satisfaction was either highest or lowest.

Qualitative information in the PJM was divided into two key aspects. First, it distinguished the factors that enhance the patient’s experience from those that detract from it; and second, it categorized the hospital’s strengths and areas for improvement. Additionally, quantitative results were presented as mean values, providing a clear, objective measure of patient satisfaction. Preliminary PJM, incorporating both qualitative themes and quantitative results, were presented to participants and key stakeholders for further validation and discussion.

## 3 Results

### 3.1 Stages of the patient journey

The hospitalization process was structured into five sequential stages (1): Start of Hospitalization (emergency waiting, scheduled admission wait, ward admission) (2), Communal Living (nocturnal rest, awakening, hygiene/shower routines, meals) (3), Medical Process (medication administration, medical visits) (4), Non-Pharmacological Treatment (morning activity, afternoon activity, door opening, therapeutic walks, family visits), and (5) End of Hospitalization (discharge process). These stages were validated through focus group feedback and expert panel consensus, forming the framework for analyzing patient experiences.

### 3.2 Qualitative findings

The analysis of patient journey experiences identified a combination of positive elements and areas needing improvement throughout the hospitalization process. Many patients appreciated the attentive and compassionate care from staff, as reflected in comments such as, “They were always checking on me,” and, “They made me feel at ease right away.” Timely and clear communication before scheduled admissions was also valued: “They informed me in advance about my admission date,” and, “Staff were always kind when explaining things.” Conversely, prolonged waiting times and uncertainty, especially during admission from the emergency department, negatively affected patients: “I waited for hours without knowing what to expect,” and, “No one explained where I would be admitted.” Restrictions on personal belongings and strict security measures were often described as excessive: “I would have liked to have my phone,” and, “The security measures felt too strict.” Some patients reported feeling disoriented due to insufficient information about daily routines and unit rules: “I was confused about what I could do and the daily schedules,” and, “I didn’t know if I could wear my own clothes or not.

**Figure 1:**
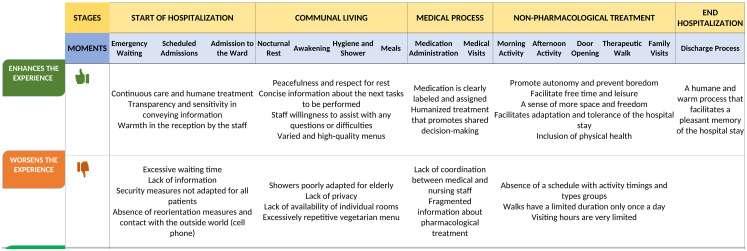
Patient-related issues presented in the PJM: factors that enhance or worsen the patient experience at each moment of the hospitalization journey (qualitative findings).

**Figure 2:**
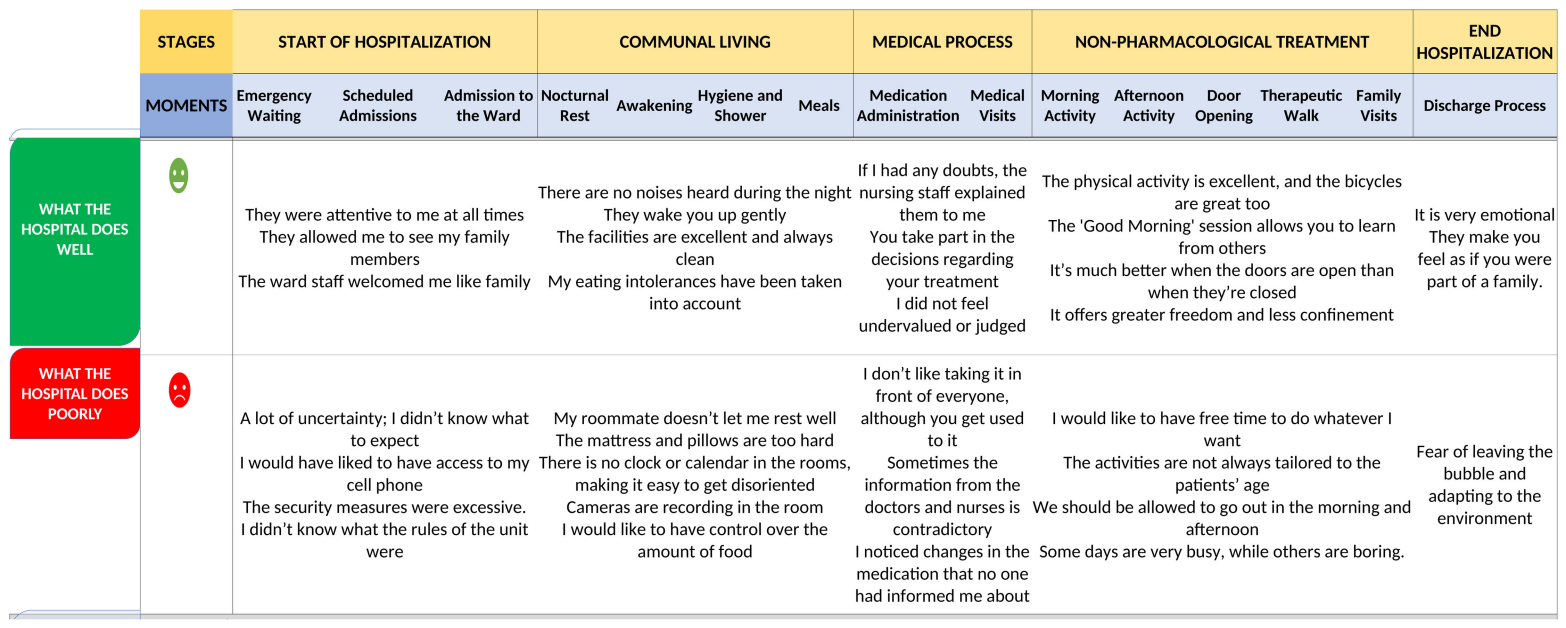
Hospital matters represented in the PJM: patients’ perceptions of what the hospital does well and what it does poorly at each moment of the hospitalization journey (qualitative findings).

Other frequently mentioned challenges included limited family visitation, discomfort related to room sharing and lack of privacy, and inconsistent information from medical and nursing teams. Despite these issues, patients consistently valued the warmth of staff, the cleanliness and modernity of facilities, and the availability of therapeutic activities. Overall, patients highlighted the need for clearer information, more flexible rules, and enhanced emotional support to further improve the hospital experience. In the PJM, these results are represented in two sections: first, patient-related issues — the factors that enhance or worsen the experience at each moment (**Figure 1**) — and second, hospital matters — what the hospital does well and what it does poorly at each moment (**Figure 2**).

**Figure 3:**
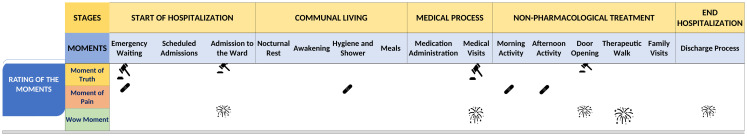
Critical touchpoints of the patient journey: Moments of Truth, Moments of Pain, and Wow Moments.

Critical touchpoints were identified and categorized as (1): Moments of Truth (emergency waiting, ward admission, medical visits, door opening), where trust and collaboration shaped perceptions (2); Moments of Pain (emergency waiting, hygiene and shower routines, and the morning and afternoon activity sessions), marked by frustration, low perceived control, or under-stimulation; and (3) Wow Moments (ward admission, medical visits, door opening, therapeutic walks, and discharge), where patient expectations were exceeded through shared decision-making and autonomy promotion. These classifications correspond to the symbols shown in **Figure 3**.

### 3.3 Quantitative outcomes

**Figure 4:**
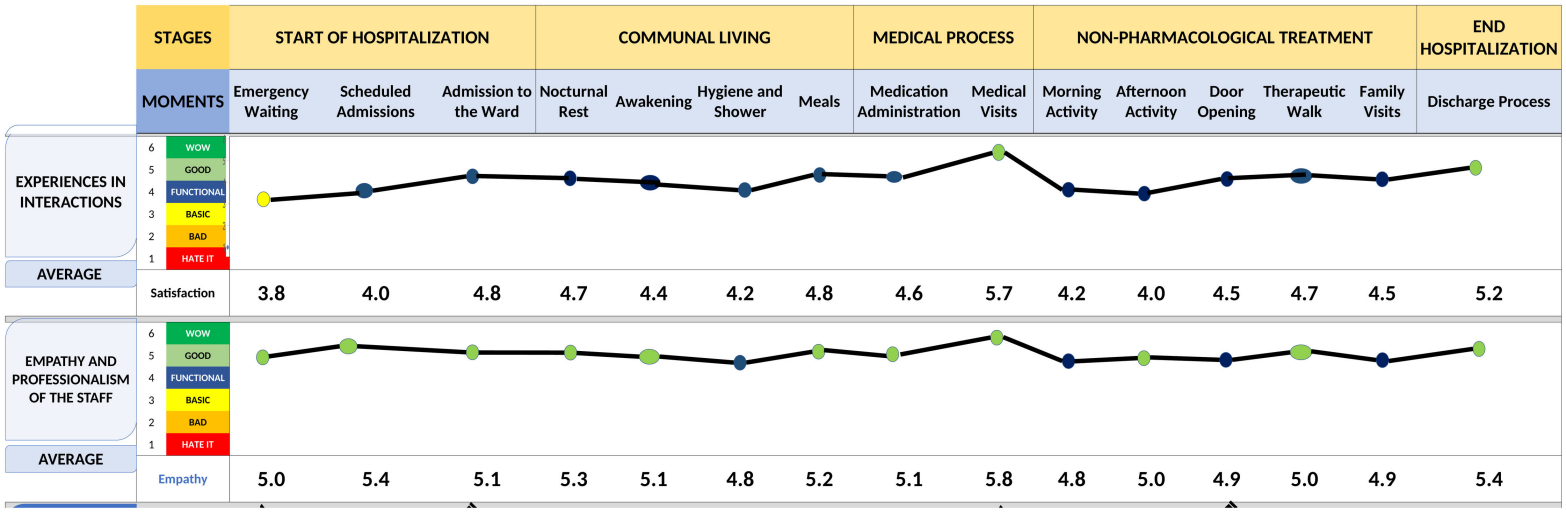
Quantitative results presented in the PJM: mean patient ratings across the journey for the two subscales (Experience in Interactions; Empathy and Professionalism of Staff).

Mean satisfaction scores (1–6 Likert scale) varied across stages (these differences are descriptive only, as no inferential tests were performed): the lowest interaction scores occurred during emergency waiting (3.8 ± 1.2) and afternoon activities (4.0 ± 0.9), while the highest scores were linked to medical visits (interaction: 5.7 ± 0.6; empathy: 5.8 ± 0.4) and discharge processes (empathy: 5.4 ± 0.7). Medication administration scored moderately (4.6 ± 1.1), reflecting discomfort with public routines, whereas communal living aspects like meals (4.8 ± 0.8) and nocturnal rest (5.3 ± 0.7 for empathy) received favorable ratings. These ratings are also displayed in the PJM, as shown in **Figure 4**.

### 3.4 Patient journey map

**Figure 5:**
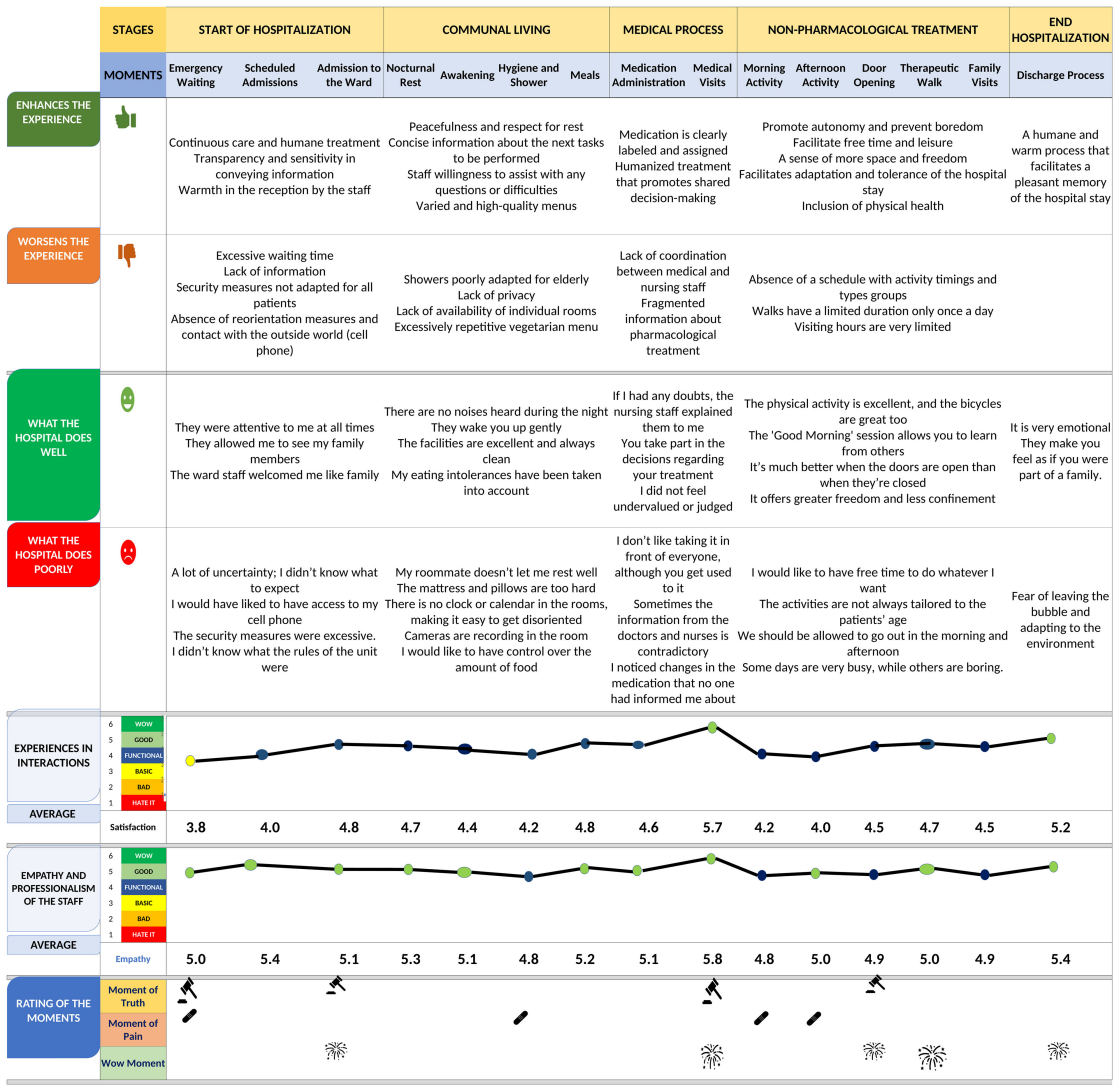
Final integrated Patient Journey Map combining qualitative findings, quantitative ratings, and critical touchpoints.

The final PJM, incorporating the study results, is presented in **Figure 5**. From top to bottom, it outlines, for each moment, factors that “Enhance” or “Worsen” the experience as well as the strengths and weaknesses of the hospital’s performance. It also provides the obtained ratings for Interaction and Empathy/Professionalism, helping to identify both strengths and areas needing improvement throughout the hospitalization process. In the bottom section, the key moments are represented using the symbols shown previously.

## 4 Discussion

This study used Patient Journey Mapping to examine the hospitalization experience in an acute psychiatric unit operating a structured, time-limited open-door policy. By integrating focus group narratives with satisfaction ratings across five predefined hospitalization stages, the results reveal a consistent pattern: patient experience is shaped less by clinical interventions per se than by the informational, relational, and environmental conditions in which those interventions take place. Stages associated with high perceived autonomy, clear communication, and empathic interaction (medical visits, therapeutic walks, discharge) received the highest satisfaction scores and the most positive qualitative appraisals, whereas stages marked by uncertainty, loss of control, and restricted agency (emergency waiting, medication routines) yielded the lowest. This convergence between qualitative and quantitative data strengthens confidence in the findings despite the exploratory nature of the quantitative instrument.

The preadmission phase, particularly emergency department waiting, emerged as the most problematic segment of the patient trajectory, with the lowest mean interaction score (3.8 ± 1.2) and recurrent qualitative themes of disorientation, helplessness, and informational deprivation. These findings are consistent with the broader psychiatric literature documenting the adverse psychological impact of prolonged emergency department boarding on individuals in acute mental health crisis (21, 30). Importantly, patients did not primarily complain about the duration of waiting itself but about the absence of structured communication during that period. This distinction has practical implications: even when systemic constraints prevent shorter wait times, regular informational updates, delivered in person or through visual displays, could attenuate the perception of abandonment. The literature on procedural justice in coercive psychiatric settings supports this interpretation: patients tolerate adverse circumstances better when they understand the process and feel acknowledged (1, 31). In contrast, ward admission itself was rated favorably, suggesting that the transition from emergency to inpatient care functions as a restorative moment when handled with empathy—a pattern consistent with findings from other European open-door units (9, 32).

Medical visits received the highest satisfaction scores across both subscales (interaction: 5.7 ± 0.6; empathy: 5.8 ± 0.4), with patients consistently highlighting experiences of being listened to and included in treatment decisions. This aligns with a substantial body of evidence linking shared decision-making in psychiatric inpatient settings to greater treatment engagement, perceived dignity, and therapeutic alliance (3, 20). By contrast, medication administration received moderate scores (4.6 ± 1.1) and was one of the stages most frequently coded as problematic in the qualitative analysis. The central issue was not pharmacological but environmental: patients objected to the public, communal nature of medication routines, which they experienced as an intrusion on dignity. This finding resonates with qualitative syntheses of restrictive practices in acute psychiatry, where loss of privacy during routine care activities is repeatedly identified as a source of distress independent of the clinical appropriateness of the intervention (4, 33). A simple organizational change, offering a semi-private space for medication administration, could address this concern without altering clinical protocols, though the feasibility of such modifications in a 14-bed unit with limited physical infrastructure warrants careful assessment.

The open-door regime was among the most positively evaluated aspects of hospitalization. Patients described the scheduled door openings as a source of autonomy, psychological relief, and normalisation. The structured group-activity program, however, presented a markedly different picture, which we consider in detail below. These findings are broadly consistent with the literature on open-door policies, which has demonstrated sustained reductions in seclusion rates (9), maintained safety standards in randomised designs (10), and positive patient perceptions of autonomy (12). However, a critical interpretive distinction must be drawn: the model implemented in the study unit, a structured 6-hour daily opening with movement restricted to the hospital floor, differs substantially from the unrestricted open-door policies evaluated in most of the cited evidence. In the Norwegian trial by Indregard et al. (10), for instance, doors remained continuously unlocked during the intervention period. Similarly, the Swiss longitudinal data reported by Hochstrasser et al. (9) and Jungfer et al. (8) derived from units with full open-door implementation. The present findings therefore suggest that even a partial, time-delimited open-door regime may generate meaningful benefits in patient experience, but the magnitude and nature of these benefits may not be directly comparable to those reported in settings with unrestricted policies. Future research should examine whether a dose-response relationship exists between the extent of door openings and patient-reported outcomes, as this has implications for units that cannot implement full open-door models due to regulatory, structural, or safety constraints.

A more critical reading is warranted for the structured activity program. Although the open-door regime was rated highly, the morning and afternoon activity sessions received interaction scores among the lowest of the entire journey (4.2 and 4.0, respectively) and were classified as Moments of Pain. This dissociation suggests that what patients valued about the ward was driven more by freedom of movement and individualised clinical contact than by the group activity program itself. The qualitative data point to two contributing factors: activities were not consistently tailored to patients’ age or interests, and their therapeutic rationale was not made explicit, so that some patients appeared to experience them as time-filling rather than as integral components of their treatment and recovery. It is notable that the highest-rated touchpoint, medical visits, was also the most individualised, which may indicate that opportunities for personal agency and formulation-based engagement were concentrated in a small part of the day. These observations echo concerns in the literature that inpatient activity provision can default to occupation rather than recovery-oriented, co-produced intervention. Reconfiguring the program around patient choice, age-appropriate and goal-directed activities, and explicit links to each patient’s formulation and recovery plan could convert a current Moment of Pain into a therapeutic asset, with potential downstream benefits for engagement and, ultimately, readmission. We note, however, that the present study assessed activities only from the patient perspective and did not directly measure their therapeutic content or fidelity; these interpretations are therefore best regarded as hypothesis-generating and a priority for future, multi-stakeholder evaluation.

A notable gap identified in this stage was the inconsistency in scheduling and communication of therapeutic activities. Patients reported not knowing what activities were planned or when changes occurred, a finding that contrasts with the otherwise positive qualitative evaluation of the activities themselves. This dissociation between content satisfaction and informational satisfaction recurs across multiple stages of the journey and may represent the single most actionable improvement opportunity identified by the study: a systematic protocol for daily activity communication, achievable through low cost solutions such as updated physical boards, could enhance predictability without requiring additional resources.

The discharge process received the highest empathy scores (5.4 ± 0.7), with patients describing the interactions as warm, clear, and reassuring. This is a noteworthy finding in a context where discharge from acute psychiatric inpatient care is frequently associated with anxiety, ambivalence, and perceived abandonment in the literature (34, 35). The qualitative data suggest that the unit’s discharge practices effectively addressed informational needs and emotional preparation, likely reflecting the influence of the Safewards model’s emphasis on structured transitions (22).

Nonetheless, some patients expressed apprehension about post-discharge adaptation, a concern well documented in the psychiatric discharge literature and associated with increased readmission risk (34). Strengthening post-discharge continuity through structured follow-up contacts, telephone check-ins within 72 hours, coordination with community mental health teams, has been shown to reduce early readmission and improve subjective recovery (36), and could complement the unit’s already strong discharge process.

From a methodological standpoint, this study contributes to the growing but heterogeneous literature on Patient Journey Mapping in healthcare (14, 17, 18). The application of PJM to the psychiatric inpatient trajectory is, to our knowledge, novel in the published literature; while Wolff et al. (21) used journey mapping to examine emergency department boarding of child and adolescent psychiatric patients, no prior study has mapped the full hospitalization trajectory in an adult acute psychiatric unit. The exploratory sequential mixed-methods design adopted here, in which qualitative findings informed the construction of the quantitative instrument, aligns with recommended practices for PJM research (16) and represents an advance over single-method applications. However, the approach also exposed a methodological tension inherent to PJM: the classification of critical moments (Pain, Truth, Wow) required integrative judgement across qualitative and quantitative data sources, and the criteria for this integration, while specified in the Methods, inevitably involve a degree of subjectivity that formal mixed-methods integration frameworks such as joint displays (37) could help mitigate in future iterations.

Several limitations of this study must be acknowledged. First, the single-site design restricts the generalizability of findings; the unit’s specific characteristics, including the Safewards model implementation, the 6-hour open-door schedule, a 14-bed capacity, and the Spanish public hospital context, may not be representative of other acute psychiatric settings. Because the unit operated a structured, time-limited open-door policy (6 hours/day, movement restricted to the hospital floor) rather than a continuous open-door regime, the patient-experience benefits observed here should not be assumed to be equivalent in magnitude to those reported for fully unrestricted policies. Second, the convenience sampling approach in both phases means that the most acutely unwell patients, including those with active psychotic symptoms or severe cognitive disorganization, were systematically excluded, likely resulting in a more favorable picture of the hospitalization experience than would be obtained with a fully representative sample. Relatedly, although 60% of the included participants had been admitted involuntarily, participation required a degree of clinical stability, so the patients willing and able to take part are likely to have over-represented less acutely unwell individuals and, plausibly, voluntarily admitted patients relative to the unit’s overall case mix; both factors probably bias the satisfaction estimates in a favorable direction. This is a common limitation in experiential research in acute psychiatry (19) but should be borne in mind when interpreting the satisfaction scores. Third, the quantitative instrument was purpose-built for this study and has not undergone formal psychometric validation; the scores should therefore be treated as exploratory indicators rather than as validated measures of satisfaction. Fourth, no inferential statistical comparisons were performed between hospitalization stages; the quantitative data are purely descriptive, and statements about differences in satisfaction across stages should be interpreted accordingly. Fifth, the study captured only the patient perspective; the experiences and perceptions of nursing staff, psychiatrists, and family members were not systematically assessed, limiting the comprehensiveness of the journey map. Incorporating multi-stakeholder perspectives is a recommended standard in PJM research (14, 16) and should be prioritized in future applications.

Future research should address these limitations by replicating the methodology in multiple sites with varying open-door configurations, incorporating validated patient-reported outcome and experience measures (PROMs/PREMs) alongside the PJM approach, and extending the mapping framework to include staff and family perspectives. Longitudinal designs that track whether PJM-identified improvements translate into measurable changes in clinical outcomes, such as readmission rates, coercive events, or patient-reported recovery, would strengthen the evidence base for this methodology. The development of a standardized PJM protocol for acute psychiatric settings, with predefined integration criteria and reporting standards, could also help consolidate a currently fragmented field (18).

## Data Availability

The raw data supporting the conclusions of this article will be made available by the authors, without undue reservation.
